# Using funnel plots in public health surveillance

**DOI:** 10.1186/1478-7954-9-58

**Published:** 2011-11-10

**Authors:** Douglas C Dover, Donald P Schopflocher

**Affiliations:** 1 Alberta Health and Wellness, Edmonton, Canada; 2 School of Public Health, University of Alberta, Edmonton, Canada

## Abstract

**Background:**

Public health surveillance is often concerned with the analysis of health outcomes over small areas. Funnel plots have been proposed as a useful tool for assessing and visualizing surveillance data, but their full utility has not been appreciated (for example, in the incorporation and interpretation of risk factors).

**Methods:**

We investigate a way to simultaneously focus funnel plot analyses on direct policy implications while visually incorporating model fit and the effects of risk factors. Health survey data representing modifiable and nonmodifiable risk factors are used in an analysis of 2007 small area motor vehicle mortality rates in Alberta, Canada.

**Results:**

Small area variations in motor vehicle mortality in Alberta were well explained by the suite of modifiable and nonmodifiable risk factors. Funnel plots of raw rates and of risk adjusted rates lead to different conclusions; the analysis process highlights opportunities for intervention as risk factors are incorporated into the model. Maps based on funnel plot methods identify areas worthy of further investigation.

**Conclusions:**

Funnel plots provide a useful tool to explore small area data and to routinely incorporate covariate relationships in surveillance analyses. The exploratory process has at each step a direct and useful policy-related result. Dealing thoughtfully with statistical overdispersion is a cornerstone to fully understanding funnel plots.

## Background

According to a widely cited definition proposed by the CDC, "Public Health Surveillance is the on-going, systematic collection, analysis, and interpretation of health data essential to the planning, implementation, and evaluation of public health practice, closely integrated with the timely dissemination to those who need to know" [1]. The results of analyses conducted on data collected within a surveillance system can be used to inform public health policy and planning, to monitor the health status of a population, and to stimulate research. A functional surveillance system will provide information about the number of health events of specified types that occur within specified populations on an ongoing basis and can therefore be used to derive disease and health event rates over time in different areas (or subpopulations of other types).

One routine surveillance activity may be to monitor rates of disease occurrence in small areas in order to identify anomalies that might have a geographic basis and to enable the reporting of such anomalies to authorities in these areas. Substantial variability in population sizes in small areas introduces some challenges in the comparisons of rates, however, because the precision of estimation of these rates depends on the size of the population over which they are measured.

Several graphical procedures have been proposed for displaying small area rates to support the location of anomalous patterns. League plots [2] and choropleth maps [3] are two common approaches. League plots display observed rates (with confidence intervals) ordered by those rates. These plots are difficult to interpret [4] because they encourage interpretation as a rank ordering, and rank orderings are known to have extremely poor statistical properties [see for example, [5,6]]. Choropleth maps of rates apply differential color schemes to chosen categorizations (often quintiles) of observed rates and color each area on a map according to the category of its observed rate. These are also easy to misinterpret because the map reflects geographic area rather than population density and because the same data may result in maps with very different appearances, since the choice of category is arbitrary. Cartogram versions [7] attempt to redraw areas in proportion to populations but are often difficult to reconcile to geographies and still suffer from the arbitrary category problem.

Funnel plots are an alternative to both league plots and choropleth maps. Funnel plots are a form of scatter plot in which observed area rates are plotted against area populations. Control limits are then overlaid on the scatter plot. The control limits represent the expected variation in rates assuming that the only source of variation is stochastic. The control limits are computed in a fashion very similar to confidence limits and exhibit the distinctive funnel shape as a result of smaller expected variability in larger populations.

Funnel plots were first introduced in meta-analyses, where they are often used to determine whether a lack of a particular type of published findings demonstrates the presence of a publication bias [8,9]. This would be indicated by the absence of points in a particular region of the funnel (especially an absence of studies with a small sample size and a negative result).

The funnel plot can also be considered a form of control chart [2]. Control charts monitor whether a manufacturing or business process is under control. If analysis indicates that the process is currently stable, with only stochastic variation, then data from the process will vary within known limits and can be used to predict the future performance of the process. If the chart indicates that the data from the process being monitored are too variable, analysis of the chart can help determine the sources of variation, which might then be eliminated to bring the process back into control. In a funnel plot, if rate variation is only random and stochastic, then an appropriate proportion of the points representing area rates will tend to fall within the funnel, and importing control chart terminology, we might consider the (rate generation) process to be "under control." We can also revert to statistical terminology and note that the model fit is adequate (where, in this simple case, the model is of a single stable rate). When many rates fall outside the funnel, the plot can be described as "overdispersed," and it can be said that the process is not in control or the model does not fit the data well. Control chart terminology has been adapted to health system performance in various jurisdictions where it is assumed that managers within a health system can exercise control over a health event-related process [10]. Many of the issues in institutional performance monitoring are shared by health surveillance in support of public health. Both activities deal with small domains, highly variable rates, large differences in population sizes, multiple testing issues, ongoing monitoring activities, and dissemination of results to interested parties invested with the authority or responsibility to effect change.

It should also be noted that funnel plots are not limited to representing the model of a single stable rate; more complex models can underlie the estimation of the rate or quantity of interest [11]. For example, plotted rates can represent the residuals that remain after a rate, predicted from the values of relevant covariates using a regression model, has been subtracted from the observed rate. In health services research this process is typically called risk adjustment [2,11,12].

An ideal model for routine monitoring in health surveillance would begin with a model that fit the data, that is, where the rate generation process could be considered to be under control. Subsequent monitoring over time could focus on whether the rate generation process could be considered to be remaining under control. As well, funnel plots provide a natural, graphical method of assumption checking and model diagnostics during the model development process itself. At any stage, funnel plots may also locate areas with unusually high or low rates (outliers) and this might justify further field epidemiologic or research investigations.

In this paper we demonstrate the use of funnel plots for model development using motor vehicle mortality data in Alberta, Canada. We begin by constructing a funnel plot under the simple model of a single provincial rate and observe that it shows overdispersion. Then we demonstrate a risk adjustment process that largely eliminates this overdispersion. Finally, we discuss steps that emerge from the model that might be taken by public health decision-makers and discuss its use for routine monitoring.

We will speak in terms of small geographies, counts, and rates, and comparisons to an overall rate as these terms are commonly used in health surveillance. However, it should be noted that funnel plots are quite general and can be used for any domain where multiple estimates have been made using varying sample sizes.

## Methods

### Data

Data are from the province of Alberta, Canada. Alberta is located in Western Canada and has a population of 3,600,000. The province maintains a publicly-funded universally-available health care system. All residents of the province (except the military, the Royal Canadian Mounted Police, and federal inmates) are registered with the Alberta Health Care Insurance Plan (AHCIP). This Stakeholder Registry contains demographic information including addresses and therefore provides a source of population estimates by temporal and spatial boundaries.

Maps are based on the Alberta Regional Health Authorities (RHA), reflecting boundary changes introduced in December 2003 and in force until 2009. The small areas analyzed are 70 subregional boundaries created specifically for the analysis of health data [13]. RHA officials were engaged in the process to insure that the subregions would have operational relevance. A population of 20,000 was chosen as a minimum target within each subregion in order to ensure that rates would be relatively stable and this target was met in almost all cases.

The Alberta Vital Statistics Death Registry provides demographic information about each death in Alberta as well as the cause of death according to International Classification of Diseases, 10^th ^revision (ICD-10) codes. The current analysis reports motor vehicle traffic death rates during 2007. Motor vehicle traffic deaths were identified as ICD-10 codes V30-V89 with .5, V39-V79.4, V86.00, and V86.08.

Covariates for risk adjustment (seat belt use; drinking and driving; road type and utilization) are derived from the 2007 cycle of the Canadian Community Health Survey (CCHS), a self-report survey administered annually to approximately 65,000 Canadians (5,000 Albertans) by Statistics Canada [14]. Provincial health ministries are granted special access to location information for respondents in the CCHS sharefile, making it possible to estimate rates at the subregion level by linking CCHS postal codes to subregion boundary files and utilizing the CCHS survey weights.

*Drinking and driving *is the self-reported proportion of respondent drivers having driven a vehicle after two or more drinks; *seat belt use *is the self-reported proportion of drivers "always" wearing seat belts or passengers "always" wearing seat belts while in the front seat. A proxy for *road type and utilization *was based on the Statistics Canada Metropolitan Influence Zone (MIZ), a measure of the influence a major urban center has upon outlying areas substantially based upon the percent of the population that commutes daily to an urban center. Subregions were assigned the modal MIZ score.

The population, mortality, and survey data are all aggregated and analyzed at the 70 subregional boundaries.

### Funnel plots

The funnel plots use binomial control limits given by $\hat{p}\pm \Phi \left(1-\alpha ∕2\right)\sqrt{\frac{\hat{p}\left(1-\hat{p}\right)}{n}}$ where Φ(•) is the cumulative inverse normal distribution evaluated for 1-*α*% control limits. Other methods for control limit generation could be used, see [11] for a comprehensive review. To emphasize, $\hat{p}$ is fixed at the overall provincial rate as estimated from the data and *n *varies freely. The rate for each subregion is then overlaid on the plot at their actual population size and rate.

The funnel plot control limits are set at 95% and 99.8%. These correspond conceptually to the 95% confidence level often used in health services research and to the 3-sigma limits commonly used in process control.

Funnel plots for survey-based measures require a slight modification to account for the complex survey design. The population values are scaled by the particular survey question design effect to account for the additional variability due to the complex survey design [15].

### Funnel plot principles for mapping

Funnel plots are adapted to mapping through the use of z-scores [3,16]. The funnel plot based z-scores are computed as $\frac{p_{i}-\hat{p}}{\sqrt{\frac{\hat{p}\left(1-\hat{p}\right)}{n_{i}}}}$ where $\hat{p}$ is the provincial rate, *p_i _*is the *i^th ^*subregion rate, and *n_i _*is the *i^th ^*subregion population. Values greater than 2 are color-coded orange, values greater than 3 are red, values less than -2 are green, and values less than -3 are dark green. All other values are color-coded yellow. These z-score cut-offs correspond to the 95% and 99.8% control limits in the funnel plot.

### Risk adjustment

Risk adjustment was carried out using a judgment-based modeling procedure. Covariates that may explain between-region variability in rates were selected a priori. Poisson regression on mortality counts with a log(population) offset, a standard method for regression on rates, was carried out sequentially including demographic factors (age, sex), behavioral risk factors (seat belt use, drinking and driving) and environmental factors (proxy for road type and utilization). The adjusted rate is the product of the provincial crude rate and the ratio of observed to expected values from the relevant regression model. Poisson regression methods are not discussed in any further detail as the focus of this paper is on the use of funnel plots; other sources offer complete discussions of risk adjustment and regression methods [11,12]. Pearson goodness-of-fit statistics, in addition to the number of small areas outside the control limits, are reported at each stage in the modeling process.

All analyses were carried out in SAS 9.2. The macro code used to create the funnel plots is freely available from the authors.

## Results

**Figure 1:**
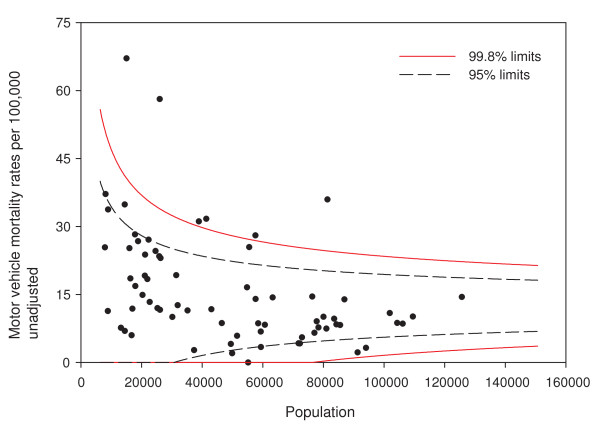
**Funnel plot of motor vehicle traffic mortality rates**.

Figure 1 shows the funnel plot of crude motor vehicle mortality rates in Alberta in 2007. Of the 70 subregions, 16/70 (23%) fall outside the 95% funnel plot control limits and 6/70 (9%) fall outside the 99.8% limits. If the model of a single provincial rate were correct (i.e., the rate generation process assumed by the model underlying this plot were in control), a funnel plot with 95% control limits could be expected to have three or four (out of 70) areas fall outside the limits. The figure shows substantial overdispersion, and we can conclude that the model does not fit well. That is, there might be additional factors (unmeasured covariates) that differ between subregions contributing to the increased variability of rates between subregions.

In searching for a model with a better fit to the data, we begin by adjusting for demographic factors, age distribution, and sex. Then, we adjust for two well-known behavioral risk factors of motor vehicle mortality for which health surveillance data are regularly available: seat belt use and drinking and driving [17]. Finally, the model is adjusted for the proxy for road type and utilization.

**Figure 2:**
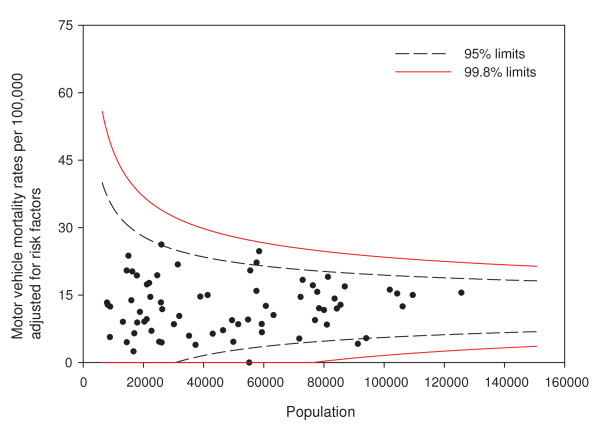
**Funnel plot of adjusted motor vehicle traffic mortality rates**. Motor vehicle mortality rates are adjusted for age, sex, seat belt use, and road type and utilization.

**Figure 3:**
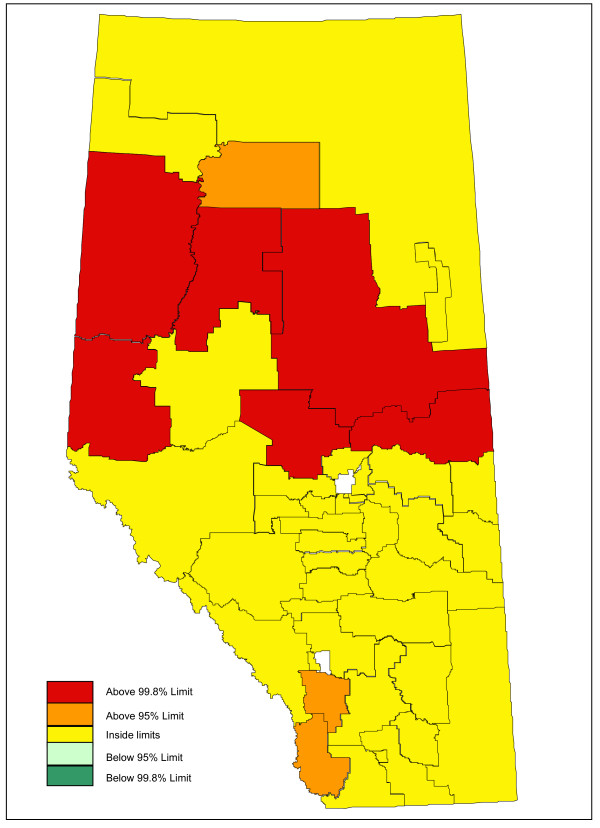
**Alberta map of unadjusted z-scores, motor vehicle traffic mortality rates**.

**Figure 4:**
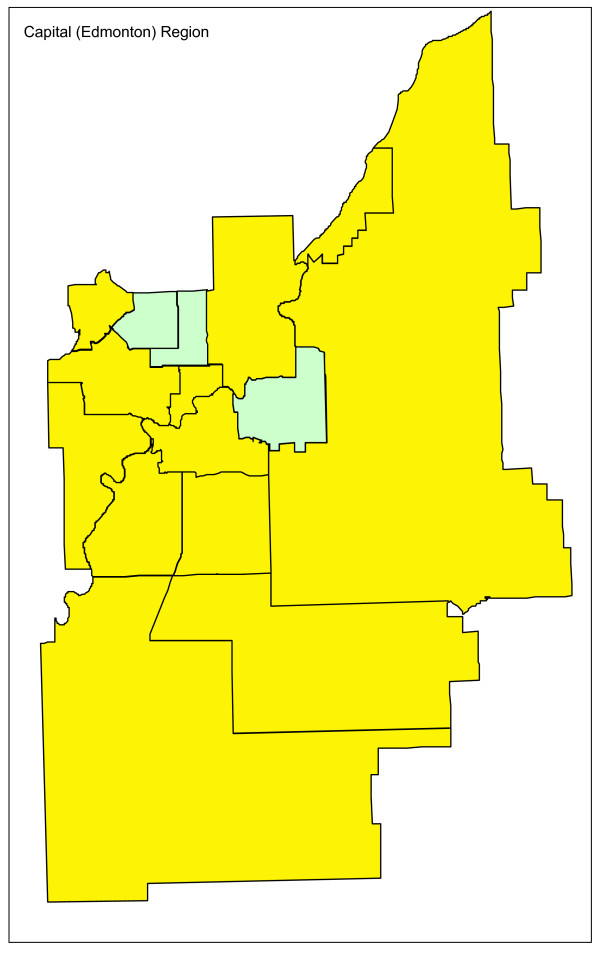
**Calgary map of unadjusted z-scores, motor vehicle traffic mortality rates**.

**Figure 5:**
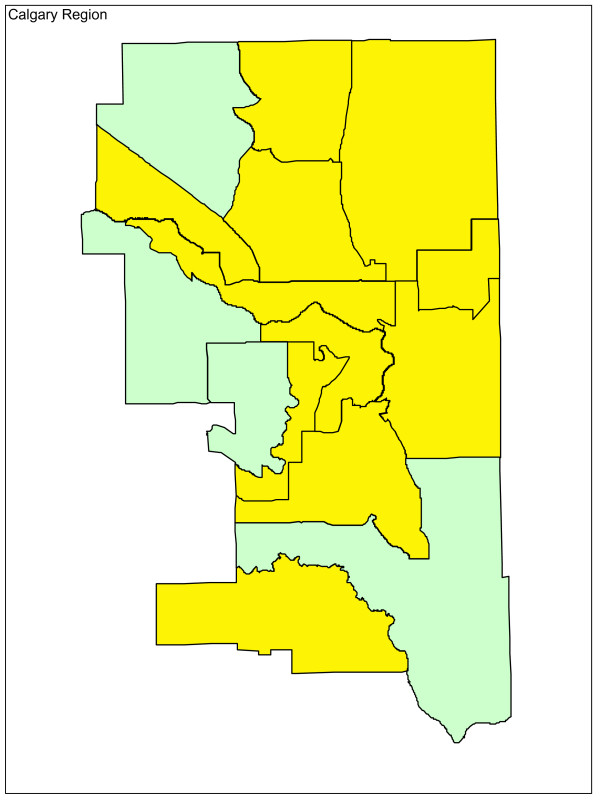
**Edmonton map of unadjusted z-scores, motor vehicle traffic mortality rates**.

**Figure 6:**
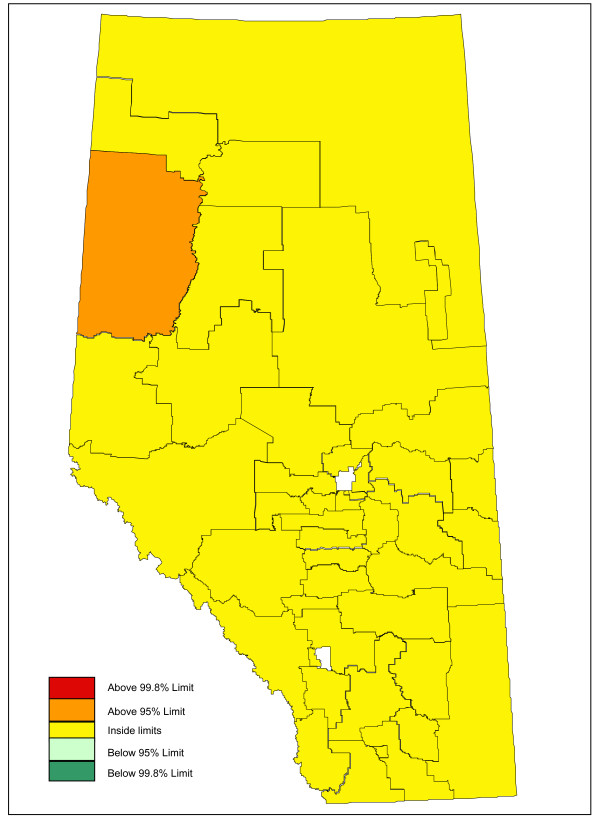
**Alberta map of adjusted z-scores, motor vehicle traffic mortality rates**.

**Figure 7:**
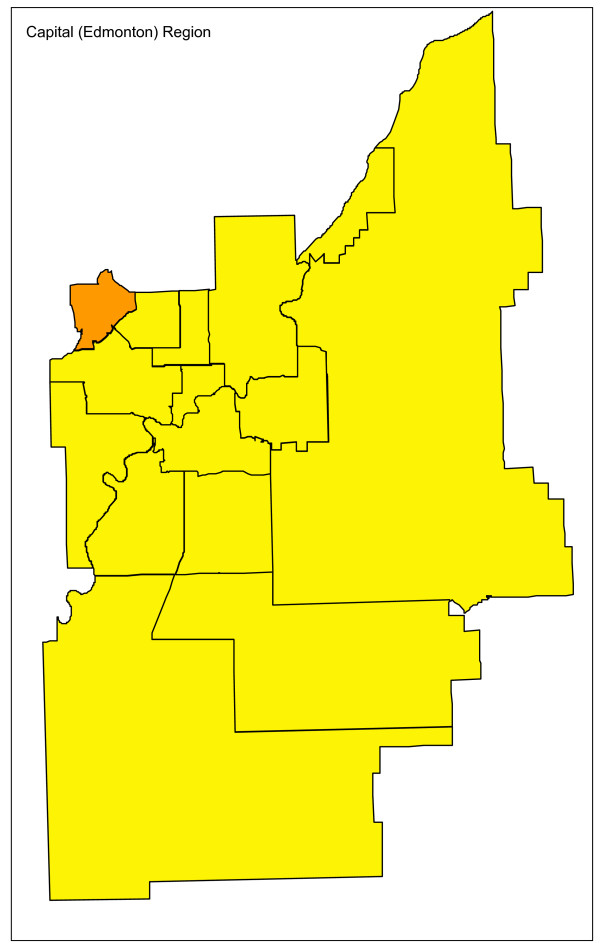
**Calgary map of adjusted z-scores, motor vehicle traffic mortality rates**.

**Figure 8:**
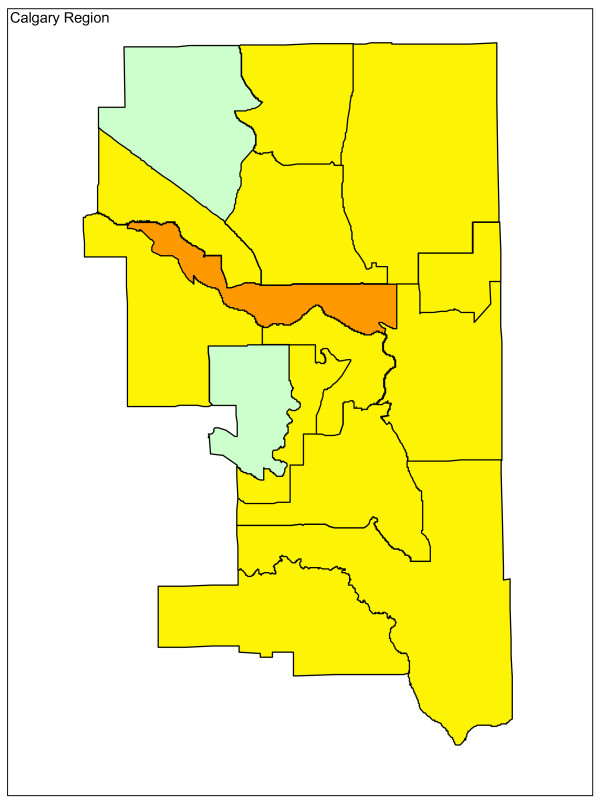
**Edmonton map of adjusted z-scores, motor vehicle traffic mortality rates**.

Adjusting for age and sex differences between small areas has improved the model fit substantially, with the Pearson chi-square goodness of fit now down to 1.55 from 3.78 in the raw data. Age distribution and sex are maintained in the model and drinking and driving is included. Drinking and driving has little effect on the model: the goodness of fit is unchanged and the model p-value for drinking and driving is not significant (p = 0.41). Removing drinking and driving from the model, the inclusion of seat belt use offers a very slight improvement in fit (goodness of fit 1.52), even though it is not significant at the alpha = 0.05 level in the model (p = 0.07). Finally, the proxy for road utilization and type is added to the model. There is a substantial improvement in fit and seat belt use is now suggestive in the model (p = 0.09). Seat belt use is maintained in the model based on a combination of its public health policy relevance and its suggestive significance level. A final model including age, sex, seat belt use, and proxy for road type and utilization is kept, with a goodness of fit of 1.34, 5/70 subregions outside the 95% limits, and no subregions outside the 99.8% control limits. A funnel plot of age-sex-seat belt use-road adjusted rates is shown in Figure 2. The sequence of models, their goodness of fit statistics, and the number of points outside the 95% and 99.8% limits is shown in Table 1. Figures 3, 4 and 5 show z-score maps of the unadjusted rates, and Figures 6, 7 and 8 show the adjusted rates based on the funnel plot methodology. Figures 3 and 6 show the entire province of Alberta. Figures 4 and 7 show expanded views of the areas within Calgary, the major population center in the south. Figures 5 and 8 show expanded views of the areas within Edmonton, the major population center in the north of the province. We note here that the unadjusted funnel plot in Figure 1 has three points, at (approximate) populations 15,000, 25,000 and 85,000, that appear to have very large rates as they lie far outside even the 99.8% limits. However, examining the adjusted funnel plot in Figure 2, these points are no longer anomalous after adjusting for age, sex, seat belt use, and road type and utilization.

**Table 1:** Modeling between subregion variation in motor vehicle traffic mortality rates

	Pearson goodness of fit	Outside 95% limits (#)	Outside 99.8% limits (#)
Unadjusted	3.78	16	6
			
Adjusting for:			
Age, sex	1.55	9	2
Age, sex, drinking and driving	1.55	8	1
Age, sex, seat belt use	1.52	7	2
Age, sex, seat belt use, road type and utilization	1.34	5	0

**Figure 9:**
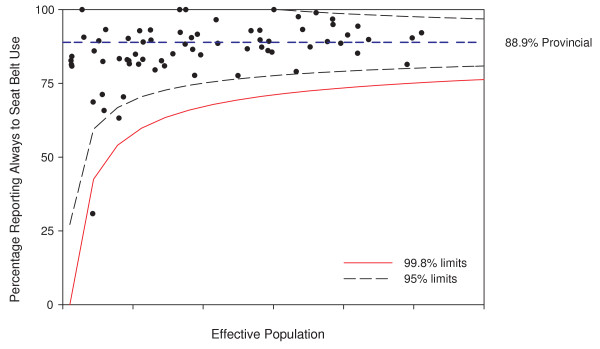
**Funnel plot of seat belt use rates**. The x-axis values have been suppressed to maintain confidentiality.

**Figure 10:**
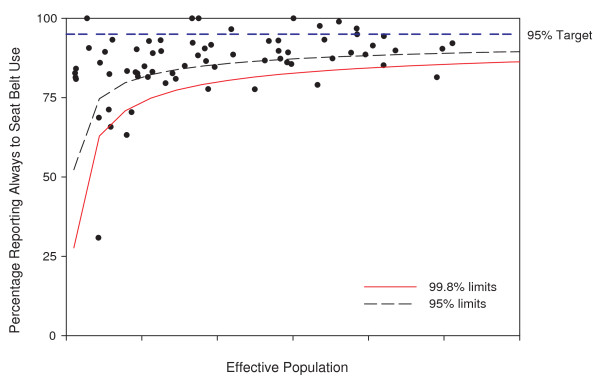
**Funnel plot of seat belt use rates centered at target 95%**. The x-axis values have been suppressed to maintain confidentiality.

Adjusting for *modifiable *risk factors, like seat belt use and drinking and driving, leads to further applications of funnel plots. Funnel plots showing these risk factors are also possible, again opening up for their use in anomaly detection and informing the focus of public health interventions or prevention activities. Figure 9 shows the application of the funnel plot to the survey results on seat belt use. This funnel plot focuses attention on areas that may be different from the provincial average seat belt use rate of 88.9%. Figure 10 shows the same points, but the funnel plot limits are adjusted to a target seat belt use rate of 95% [18] as an example. This visually emphasizes the proportion of subregions in the province reaching or not reaching the target level.

**Figure 11:**
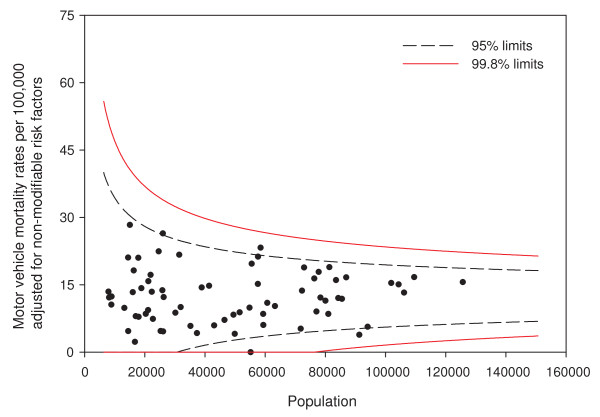
**Funnel plot adjusted for nonmodifiable risk factors**. Motor vehicle mortality rates are adjusted for age, sex, and road type and utilization.

Adjusting for *nonmodifiable *risk factors also leads to the ability to clearly explain the difference between the crude and adjusted rates, and hence the potential impact programs could have to those interested in community-level policy and interventions. For example, the crude rate of 66 per 100,000 (population near 15,000 in Figure 1) appears quite high. Figure 11 shows motor vehicle mortality rate adjusted for only the nonmodifiable risk factors age, sex, and road type and utilization. The adjusted rate for the particular subregion is now 29 per 100,000 and inside the 95% funnel plot limits. This subregion has, compared to other parts of the province, a younger, more male population and rural roads with combined effect on motor vehicle mortality rates that can be readily seen. These factors are not likely to be affected by policy or intervention.

Since the CCHS implements a complex survey design, the funnel plots have been adjusted for the design effect of 3.8 for seat belt use in 2007. All survey related points are randomly jiggled in the figures and the axis has been suppressed to protect confidentiality as required by Statistics Canada, the statistical agency that owns the data.

## Discussion

One interesting aspect of the funnel plot in Figure 1 is the substantial number of rates for small areas falling outside the funnel plot limits. This overdispersion is not an unusual phenomenon in health data [19]. The ability of the funnel plot to clearly show overdispersion is, we feel, one of the most useful aspects of the funnel plot. We can immediately and visually see that we don't fully understand the disease process. This judgment should be considerably easier than judgments of the presence or absence of publication bias when considering funnel plots of effect sizes from a meta-analysis, which depends upon the distribution of points within the funnel limits and is therefore quite error prone [20].

Funnel plots are therefore extremely useful in focusing analysts' attention on model misspecification. When overdispersion is observed, the key question becomes what to do with the apparent overdispersion. Some have advocated the use of statistical correction [21,22] to adjust for overdispersion, either through random effects models, via an overdispersion parameter, or both. We feel this should be an approach of last resort only. If there is large variability in a health variable being monitored, adjustment via the inclusion of missing covariates should be the first line of attack. We note that this adjustment need not be directly causally based. For example, if seat belt use data were not available, but a similar risk taking behavior variable was, that proxy variable could still have served to substantially explain the variability in motor vehicle mortality rates. With the plethora of survey and administrative data available today, there is no reason not to attempt to understand and model the factors affecting between-region variability before resorting to random effects-type models. Also, these blind approaches to overdispersion carry substantial risk in the surveillance arena. In the case of misspecification due to a missing covariate, random effects models make the strong assumption that the missing covariate value is essentially proportional to the observed rate [23]. It is very easy for this not to be the case in practice, as illustrated in our example. In fact, had further attempts at adjustment not been made, the interpretation of the funnel plot would have pointed public health epidemiologists to the wrong area. The purported statistical approach to fixing overdispersion must be used with great caution. Echoing Berk et al [24], "one risks an arbitrary correction leading to arbitrary results." In the motor vehicle mortality example, one small area would still have been outside the 99.8% controls limits if an overdispersion factor had been included, even though our analysis shows that this was not any sort of outlier but simply has a poor combination of age, sex, seat belt use, and road type. In the analysis presented, the final model shows good fit. Had this not been the case, it would have been possible to include random effects or an overdispersion factor in the final model. Future surveillance and monitoring efforts could continue, keeping the random effects and/or overdispersion value fixed. Attempts to dynamically alter the overdispersion parameter or re-predict random effects might only mask any real changes over time.

The funnel plot methodology encourages the use of data from multiple sources. Funnel plots in the surveillance domain can rely on aggregate data, making the linking process between data sources much easier to facilitate. In our example, we were able to seamlessly integrate survey and administrative data sources because they are only required to be available at the aggregate subregion level. This also has implications for ongoing monitoring: with systems in place to create small area estimates from a variety of data sources, ongoing monitoring and creation of future funnel plots should be possible.

Underlying the outlined funnel plot methodology is the choice of method for creating the limits. The asymptotic normal approximation was used. This choice may appear unusual in light of the fact that, for binary confidence intervals, the use of the asymptotic normal approximation is generally not recommended as it can have very poor performance characteristics [see [25] for a recent review]. Ongoing research by the authors suggests that it is the opposite case for funnel plots and the asymptotic normal approximation outperforms other methods for creating limits.

The funnel plot methodology was also successfully adapted to a mapping framework. The ability to display surveillance data in a geographic context can aid in the understanding of that data. The maps, combined with expert knowledge of the areas, can generate suggestions as to what factors may explain any residual overdispersion.

Following the institutional performance literature, funnel plots of disease rates, risk factors, or changes in these could also be used as performance measurement tools [11]. Using target rates as the funnel plot center line and placing the funnel around them gives an indication of how many small areas are likely achieving a public health target. The funnel plot of seat belt use rates around a target of 95% in Figure 10 gives a visual representation of both the range of seat belt use rates and the number of areas where seat belt use is below the target.

The recommended analysis process employing sequential funnel plots and multiple covariates lends itself to identifying opportunities for policy recommendations. For a covariate that does enter the model, there is evidence that the covariate varies across the province, naturally suggesting that further analysis of this covariate might identify local area level intervention and policy opportunities. If a known risk factor does not enter the model, a global policy level recommendation may be in order. In our example, drinking and driving did not enter the final model, suggesting that policy recommendations could be made at the provincial level; while seat belt use, which did enter the final model, lends itself to local level interventions. Further consideration of factors as modifiable or nonmodifiable facilitates the interpretation of individual small area rates. Adjusting for nonmodifiable risk factors allows a clear comparison to crude rates and highlights the potential for improvement through modifiable factors. Assessing the modifiable factors through their own funnel plots can help target local area level interventions and policy initiatives.

The use of funnel plots and modeling to assess the relationships between potential risk factors and outcomes must always be carried out with care. The process described employs an ecological model and carries with it the potential limitations and cautions of this type of design [see for example, [26]]. Particular care should be taken in interpreting the meaning of any coefficients in the model to avoid the ecological fallacy. We have framed the process as a surveillance activity where there is usually an evidential basis for inclusion of risk factors or proxies for risk factors. Clearly any single ecological correlation would be insufficient evidence to justify public health action, but when noted in the context of established risk factors, public health activities may be reasonable.

We envision three key areas for the evolution of the funnel plot in public health surveillance. The first area is the integration of the funnel plot into ongoing monitoring activities over time. We have touched on issues regarding the use of random effects and overdispersion parameters as they relate to repeated applications of a funnel plot over time. The questions related to incorporating modeling into a funnel plot-based surveillance process (Re-run the model each year with additional data? Hold coefficients constant over time? How best to display multiple years of data?) are an area of active inquiry. A related area for evolution of the funnel plot is how to appropriately incorporate funnel plots into a multilevel model framework. As multiple levels of data are becoming available for analysis in surveillance, multilevel models will become more common. Finally, funnel plots have a close link to spatial data as they are currently used in public health surveillance. The ties, theoretical and applied, to spatial methods provide a large area for future contributions.

## Conclusions

Funnel plots and their cartographic equivalents provide visually attractive means of displaying small area data in health surveillance and other disciplines for the purposes of anomaly detection and ongoing monitoring, while accounting for variation in small samples. Overdispersion, readily apparent when present in funnel plots, needs to be dealt with thoughtfully in the analysis and modeling stages of surveillance to ensure that the interpretation of the surveillance data is appropriate. The use of funnel plots in health surveillance modeling activities naturally focuses attention to the level that policy recommendations should be made.

## Competing interests

The authors declare that they have no competing interests.

## Authors' contributions

DCD and DS contributed to the design and conceptualization of the study, the interpretation of the data, and the writing of the manuscript. DCD performed the statistical analysis. All authors have approved of the final draft of the manuscript.
